# Supplementary material for: Genomic variation and epidemiology of SARS-CoV-2 importation and early circulation in Israel
Source: PLoS One. 2021 Mar 26;16(3):e0243265. doi: 10.1371/journal.pone.0243265 (PMC7997042; doi:10.1371/journal.pone.0243265)
Supplement: Attachment — Submitted filename: Response to Reviewers.docx [file pone.0243265.s002.docx]

We thank the reviewers for their comments, and attach our answers below, in red.

5. Review Comments to the Author

Please use the space provided to explain your answers to the questions above. You may also include additional comments for the author, including concerns about dual publication, research ethics, or publication ethics. (Please upload your review as an attachment if it exceeds 20,000 characters)

**Reviewer #1:** The paper entitled "Genomic epidemiology of SARS-CoV-2 importation and early circulation in Israel" presents the molecular epidemiology of SARS-CoV-2 cases over time in selected portions of Israel. Overall, the methods and analysis conducted are fairly straightforward and properly used. The findings add to the growing picture of different variant emergence globally, and would be of interest to the field.

However, the figures of the paper are cut off, so it is difficult to completely analyze the findings of the paper.

The figures will be re-submitted properly.

I also believe that the authors could expand a little more in the discussion in terms of comparison of the clade trends observed in the isolates they analyze with other countries, especially those near Israel.

We thank the reviewer for this comment. We added information regarding dominant clades similar to Israel from additional European countries (line #263). Europe and the USA are relevant to Israel in terms of viral transmission due to travel patterns, more than countries bordering with Israel, and this is the reason those were included. In addition, the paucity of data available for countries near Israel does not allow drawing clear conclusions.

Maybe the authors have included it, but a figure more directly associating clade prevalence as a function of time course of the outbreak in Israel would be of value.

This information is shown in Figure 2A.

Finally, I think some minor typos and odd phrasing in places can be corrected, though this would normally be done by a copy editor as they are minor.

We have thoroughly gone over the manuscript again to correct any typos and phrasing.

Overall, I think this would normally be something I would recommend "Minor Revisions" for, but I would encourage the authors to resubmit/clear the figures in full form (not cut off) with the Editor.

**Reviewer #2:** the paper is clearly written and well organized, the results and figures are comprehensive and helpful, please consider the highlights in the attached file for the minor reversions and justifications for questions.

We thank the reviewer for these comments. Below are our answers to the comments highlighted in the manuscript:

1. Page #1:
   title – changed to “Genomic variation and epidemiology of SARS-CoV-2 importation and early circulation in Israel” as the reviewer suggested.

   Author roles:
   Conceptualization, N.S.Z and M.M; Methodology, N.S.Z, E.B, Y.D and O.E; Validation, E.B and O.E; Formal analysis, N.S.Z, E.B and Y.D; Investigation, N.S.Z and E.B; Data curation, N.S.Z, R.P and M.M; Writing – original draft, N.S.Z; Writing – review and editing, N.S.Z, E.M, D.S, O.M and M.M; Visualization, N.S.Z; Supervision, N.S.Z, E.M, O.M and M.M; Project administration, N.S.Z, O.M and M.M.
2. Page #2:
   Abstract – the number of samples mentioned indicates the samples that were sequenced by us and described in the manuscript. Other samples included are Israeli-based samples taken from GISAID (publically available database). This is mentioned in the abstract: “with additional sequences identified Israel available in public databases”.
   In addition, we added the number of sequences we utilized from GISAID in the methods section (line 87).
3. Page #4:
   Materials and methods: we added the type of samples – nasal-pharyngeal (line 86).
   PCR: term was changed to real time PCR (RT-PCR). In our experience, the maximum Ct value that can be sequenced using the methodology described (targeted SARS-CoV-2 sequencing using the Artic primers) is ~35.
4. Page #7:
   Results: all methods-related duplicate information was deleted.

finally: I have a question about the period of sampling; why you did not conduct your research on samples till (August for example)? to cover wide range of samples to be more comprehensive about the situation.

The purpose of the manuscript (that was written back in June..!) was to describe the origin of SARS-CoV-2 in Israel and its initial spread (the “first wave”). This first wave had declined in May 2020, and a “second” wave started in July and is still ongoing. We are currently sequencing samples from that time-period to characterize the second wave.
